# FDG-PET in the diagnosis of primary progressive aphasia: a systematic review

**DOI:** 10.1007/s12149-024-01958-w

**Published:** 2024-07-19

**Authors:** Melika Mirbod, Cyrus Ayubcha, Hyae Won Kim Redden, Eric Teichner, Robert C. Subtirelu, Raj Patel, William Raynor, Thomas Werner, Abass Alavi, Mona-Elisabeth Revheim

**Affiliations:** 1 Department of Radiology, Hospital of the University of Pennsylvania, Philadelphia, PA USA; 2 Harvard Medical School, Boston, MA USA; 3 Department of Epidemiology, Harvard Chan School of Public Health, Boston, MA USA; 4 Sidney Kimmel Medical College, Thomas Jefferson University, Philadelphia, PA USA; 5 The Intervention Center, Division of Technology and Innovation, Oslo University Hospital, Oslo, Norway; 6 Institute of Clinical Medicine, Faculty of Medicine, University of Oslo, Oslo, Norway

**Keywords:** Primary progressive aphasia, PET, Positron emission tomography, FDG, 2-deoxy-2-[18F]fluoro-D-glucose, Neurology

## Abstract

Primary progressive aphasia (PPA) is a disease known to affect the frontal and temporal regions of the left hemisphere. PPA is often an indication of future development of dementia, specifically semantic dementia (SD) for frontotemporal dementia (FTD) and logopenic progressive aphasia (LPA) as an atypical presentation of Alzheimer’s disease (AD). The purpose of this review is to clarify the value of 2-deoxy-2-[18F]fluoro-D-glucose (FDG)-positron emission tomography (PET) in the detection and diagnosis of PPA. A comprehensive review of literature was conducted using Web of Science, PubMed, and Google Scholar. The three PPA subtypes show distinct regions of hypometabolism in FDG-PET imaging with SD in the anterior temporal lobes, LPA in the left temporo-parietal junction, and nonfluent/agrammatic Variant PPA (nfvPPA) in the left inferior frontal gyrus and insula. Despite the distinct patterns, overlapping hypometabolic areas can complicate differential diagnosis, especially in patients with SD who are frequently diagnosed with AD. Integration with other diagnostic tools could refine the diagnostic process and lead to improved patient outcomes. Future research should focus on validating these findings in larger populations and exploring the therapeutic implications of early, accurate PPA diagnosis with more targeted therapeutic interventions.

## Introduction

Aphasias refer to a group of language disorders that result from damage to specific areas of the brain responsible for language processing. Language impairment encompasses difficulties in speaking, understanding, reading, and writing. Fluent and nonfluent aphasias represent two broad categories of language disorders [1]. Fluent aphasias involve relatively preserved language fluency despite impaired comprehension. Wernicke's aphasia is a notable example of a fluent aphasia, characterized by fluent but nonsensical speech. Nonfluent aphasias, on the other hand, are marked by reduced speech output and effortful articulation, with intact comprehension. Broca's aphasia is a prominent nonfluent type, characterized by telegraphic speech and difficulty forming grammatically complex sentences.

Primary progressive aphasia (PPA) is a neurodegenerative disorder characterized primarily by persistent language impairment [2]. Initial symptoms often include word-finding difficulties, progressing over time to challenges in understanding language and constructing coherent sentences [1]. Disease progression may also affect planning, organization, balance, and swallowing, often necessitating caregiver support due to the resultant decline in activities of daily living. PPA can be clinically identified even while other cognitive abilities, like memory of everyday occurrences, visual and spatial aptitudes, and behavioral aspects remain reasonably functionable. Using the Gorno-Tempini criteria, a diagnosis can be made when language stands out as the primary area of impairment for at least the initial two years of the illness, and brain imaging studies, typically magnetic resonance imaging (MRI) or positron emission tomography (PET) scans, demonstrate no distinctive damage aside from atrophy that could explain the language difficulties [3]. The disease progresses in a matter of years while leaving behavioral aspects relatively intact during the early stages [4].

**Fig. 1:**
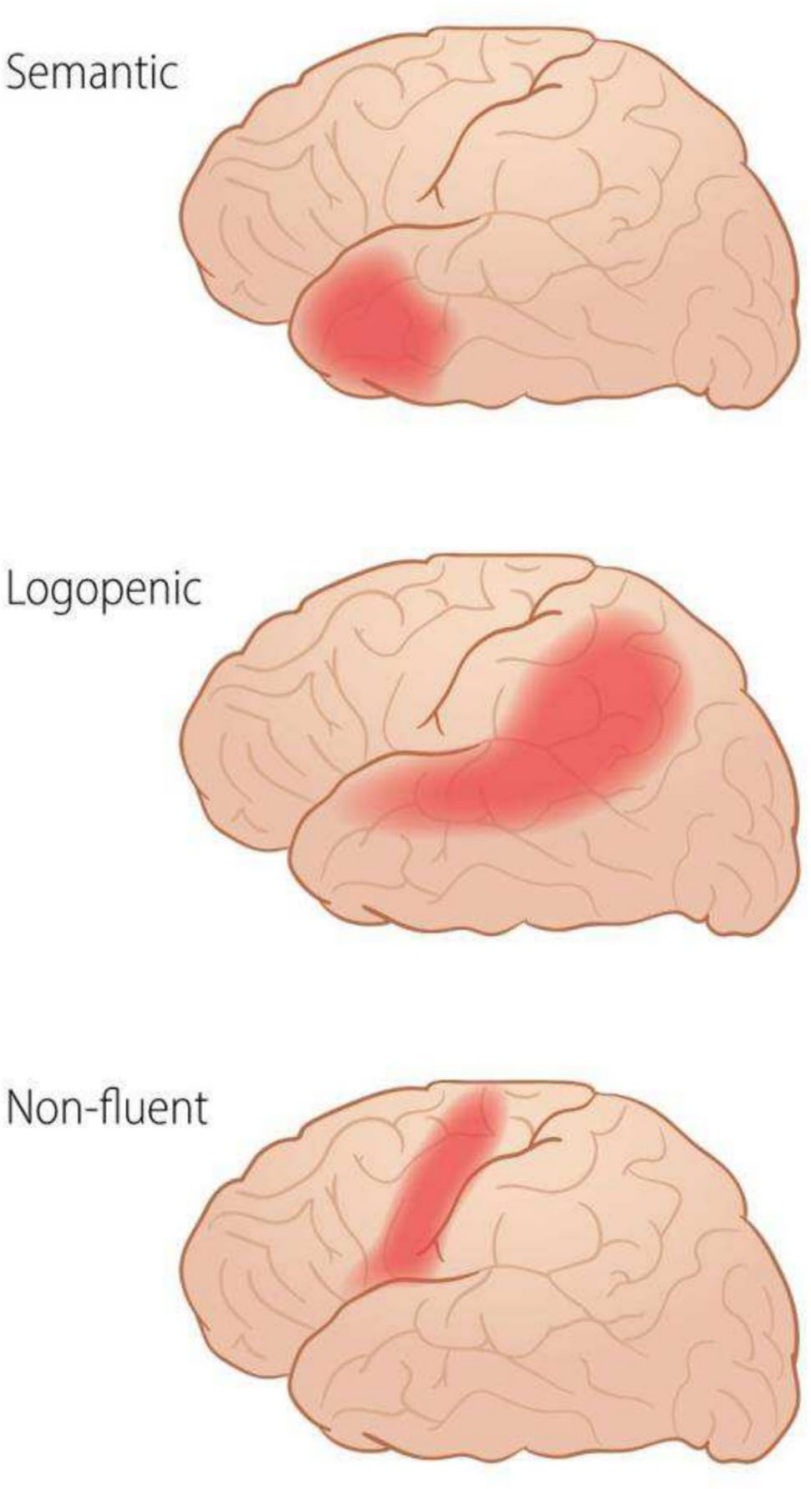
Representation of the regions of hypometabolism with the three primary progressive aphasia (PPA) variants. Reproduced with permission from Bekkhus-Wetterberg, Peter et al. [25]

Within the diagnostic framework of PPA, the condition is further subdivided into three subtypes: Semantic Dementia (SD), Progressive Non-Fluent Aphasia (PNFA), also known as the agrammatic variant (agPPA), and Logopenic Progressive Aphasia (LPA). The main regions affected in the three subtypes are shown in Fig. 1.

Common hallmarks for all subtypes include behavioral changes, sentence formation difficulties, speech comprehension issues, and naming challenges, but individual subtypes may present with unique symptomatology as the disease advances. SD commonly manifests with difficulties naming objects or thinking of words, with reduced metabolism in the anterior temporal lobe and at a later stage atrophy of the same area is typically observed in this variant. PNFA is distinguished by limited speech production and damage to the left posterior frontal and insular areas, while LPA is characterized by challenges in word finding and reduced metabolism and atrophy of the left temporo-parietal region [5].

However, existing diagnostic criteria using only these three variants may be inadequate in encompassing all individuals with PPA. An increasing body of literature now supports the likelihood of 5–6 subtypes instead of the traditionally recognized three. The newly identified subgroups exhibit more precise neuroimaging signatures, including reduced metabolism in the left frontal lobe, that better predict clinical course [6]. "Mixed dementia," often found in older populations and characterized by the presence of two or more underlying diseases, also merits consideration when there are coexisting pathologies [7].

Though the development of PPA is multifactorial, there are several risk factors, including genetic and environmental components, identified to be associated with PPA. Age, but not gender, has been identified as a significant risk factor, as PPA typically manifests between the ages of 50 and 60 but with a roughly equal prevalence among both sexes [8, 9]. Genetic predispositions, notably mutations in the granulin precursor (GRN) gene, a gene which codes for the protein progranulin, have been observed in PPA cases [10]. Most GRN mutations lead to progranulin haploinsufficiency, impacting the transactive response DNA binding protein of 43 kDa (TDP-43) gene, a protein in charge of the regulation of gene expression associated with language networks [10]. Although these genetic irregularities with the GRN gene can also be linked to other forms of dementia, further genetic research is essential to ascertain their specific association with PPA. One study highlighted a higher prevalence of learning disabilities, particularly dyslexia, among PPA patients compared to other dementia groups and controls, suggesting a potential association [11]. Environmental factors, such as injuries to the left hemisphere, may also elevate PPA risk [11]. Toxins and pesticides such as lead, aluminum, and mercury have also been linked to neurodegenerative diseases [12].

Anatomically, PPA predominantly affects the frontal and temporal regions of the left hemisphere, which govern speech and language production. Atrophy is most apparent in Broca's and Wernicke's areas, as well as the frontal, parietal, and temporal cortices. Damage typically localizes to the left hemisphere, with the right hemisphere remaining intact. Research into understanding the pathophysiology of the different PPA variants has steadily been advancing, with recent studies elucidating an association between SD and TDP-43 pathology, PNFA with primary tauopathies, and LPA with Alzheimer’s pathology. However, there are still many ambiguities in the specific mechanisms and pathways, with no clear associations. Additionally, the three subtypes appear to have more of a mixed pathology rather than distinct patterns [13].

Management of PPA includes options such as speech and language therapy to improve language preservation, and physical or occupational therapy to address movement or balance concerns [9, 14]. Current approaches provide symptomatic relief, yet there continues to be a strong need for curative therapies. Additionally, due to current limitations in treatment avenues, early diagnosis is pivotal for effective symptom management. However, diagnosing PPA is challenging due to its diverse and complex pathophysiology, often leading to frequent misdiagnoses as Alzheimer’s disease (AD). Unlike AD patients, PPA patients usually retain memory and visual processing capabilities until the disease's advanced stages [1]. However, PPA is often an early indicator of potential dementia development, with SD correlating to Frontotemporal Dementia (FTD) and LPA considered an atypical AD presentation. Early PPA identification can significantly impacts patient management and prognosis.

Given that PPA is a neurodegenerative condition leading to a gradual loss of language abilities, early diagnosis allows for timely intervention and the implementation of appropriate therapeutic strategies. Speech and language therapy can be initiated promptly to help individuals with PPA maintain and improve their communication skills. Moreover, early identification enables healthcare professionals to differentiate PPA from other forms of dementia, facilitating accurate prognosis and tailored care plans. Understanding the specific subtype of PPA at an early stage aids in the development of targeted interventions that address the unique language deficits associated with each variant. In addition, early diagnosis provides individuals and their families with the opportunity to plan, make informed decisions about care, and access support services that can enhance the overall quality of life for those affected by PPA.

Positron emission tomography (PET) using 2-deoxy-2-[18F]fluoro-D-glucose (FDG) holds a distinct advantage over other neuroimaging modalities due to its ability to provide functional information about glucose metabolism in the brain. Unlike structural imaging techniques such as computed tomography (CT) or MRI, FDG-PET reveals metabolic activity, offering insights into the functional integrity of neural tissues. This is particularly crucial in the early detection and differential diagnosis of neurodegenerative disorders, where changes in metabolic activity often precede observable structural abnormalities. Unlike amyloid PET and tau PET, which focus on detecting specific pathological proteins associated with conditions like AD, FDG-PET provides a broader assessment of cerebral glucose metabolism. This makes FDG-PET versatile for investigating various neurological conditions.

By visualizing metabolic alterations preceding atrophy, FDG-PET has been extensively applied to PPA with the intention of uncovering underlying metabolic dysfunction prior to structural changes, demonstrating its possible benefit with PPA diagnosis. This review aims to consolidate and elucidate the value of FDG-PET in detecting and diagnosing PPA.

## Materials and methods

A comprehensive systematic literature review was conducted using Web of Science, PubMed, and Google Scholar to collate retrospective and prospective studies employing FDG-PET in PPA patients. Keywords like “primary progressive aphasia,” “PET,” and “neuroimaging” were used in the search strategy in conjunction with a PICO (Population of interest, Intervention, Control, Outcome) structured question [15]. These search terms were used with the specified databases on August 29, 2023. The terms and date of search can be seen in Table 1. The comprehensive PICO question outlines the Patient population (P), Intervention (I), Comparison (C), and Outcome (O) in a research study aimed at evaluating the accuracy and diagnostic value of FDG-PET for differentiating primary progressive aphasia subtypes and distinguishing them from other neurodegenerative diseases.

**Table 1:** Depiction of the search strategies used for PICO

PICO Search Terms		Date of Search	Number of Total Results
Patient	Primary progressive aphasia, Non-fluent, agrammatic, Semantic Dementia, Progressive Non-fluent aphasia, Logopenic Progressive aphasia	August 29, 2023	98 total papers
Intervention	FDG-PET, [^18^F]Fluorodeoxyglucose		
Comparison	Standard clinical assessments, neuroimaging, Alzheimer’s Disease		
Outcome(s)	Differential diagnosis between primary progressive aphasia subtypes, distinguishing from other neurodegenerative disorders		

The question used was, “In patients with suspected primary progressive aphasia (P), is the use of FDG-PET (I) compared to standard clinical assessments (C), effective in accurately distinguishing between the subtypes of primary progressive aphasia (non-fluent/agrammatic variant, semantic variant, logopenic variant) and differentiating PPA from other neurodegenerative diseases, specifically AD and Pick's Disease (O)?”

There was not a specified sample number of patients required for each study. Full papers written only in English were included, excluding abstracts. Only articles that met the PICO criteria were included. Excluded papers were most often removed because of their focus on Alzheimer’s disease instead of PPA or their analysis of other biomarkers that did not include FDG-PET, such as amyloid PET, tau PET, and CSF biomarkers. The Preferred Reporting Items for Systematic Reviews and Meta-Analysis (PRISMA) was used as a guide for this review [16]. The quality of each included study was assessed using the critical appraisal skills programme (CASP) checklist, a 12-question measurement tool used to check relevance and trustworthiness [17].

**Fig. 2:**
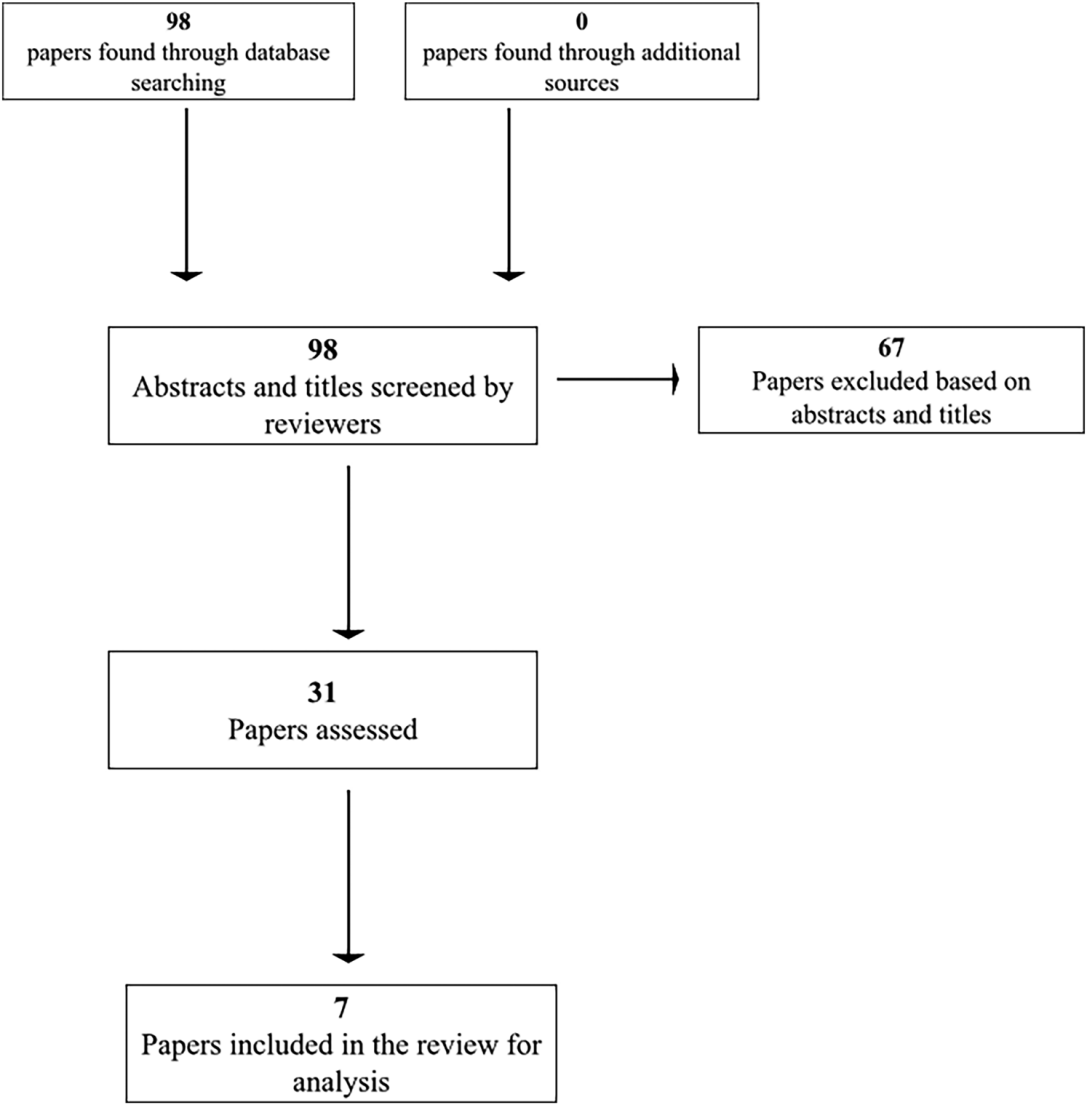
PRISMA flowchart: This diagram visually represents the flow of information throughout the different phases of the systematic review. It provides an overview of the number of identified records, including those that were included and excluded

Studies exclusively focused on Alzheimer’s disease and other biomarkers were excluded to maintain a clear and specific focus on the value of FDG-PET imaging in diagnosing and differentiating the subtypes of primary progressive aphasia (PPA). This exclusion ensures that the review remains targeted on the distinct metabolic patterns associated with PPA, thereby providing more precise and relevant insights for clinical practice in this specific context. Only studies written in English were included. These studies, published post-2000, underwent methodology quality assessment and result and outcome extraction. They encompassed peer-reviewed journals evaluating FDG-PET utility in diagnosing PPA. A diagram representing the flow of information can be found in Fig. 2. Additionally, Table 2 provides a summary and quality review of the articles included in the study.

**Table 2:** Summary of papers included with number of patients, results, tests used, FDG-PET acquisition, weaknesses of the paper, and quality review

Article Authors	Number of Patients	Results	Tests used	FDG-PET acquisition	Weaknesses	Quality Review using CASP Checklist [31]
Josephs et al. 2010 [18]	24	FDG-PET is capable of detecting early brain changes. This study utilized FDG-PET to investigate the hypometabolism patterns in various forms of PPA, with a particular focus on individuals with AOS and those with PFA	Short Test of Mental Status at time of PET scan, Minnesota Test for Differential Diagnosis of Aphasia, the Boston Naming Test (BNT), part V of the Token Test, and a letter/word fluency task	629 MBq of FDG (range, 555–740 MBq). After a 25–30 min uptake period, the patient was positioned on the scanner bed	Control subjects were not used in this study	Medium
Rabinovici et al. 2008 [22]	15	The patterns of FDG uptake exhibited focal variations depending on the subtype of PPA. Specifically, individuals with Logopenic PPA (LPA) showed reduced metabolism in the left temporoparietal region, those with Progressive Nonfluent Aphasia (PNFA) exhibited decreased metabolism in the left frontal area, and Semantic Dementia (SD) patients displayed reduced metabolism in the left anterior temporal region. Moreover, there was a significant asymmetry in FDG uptake, with a preference for left-sided hypometabolism, in individuals with PPA (*p* < 0.005)	Battery of speech and language tests to determine PPA subtype, Western Aphasia Battery (WAB), Boston Diagnostic Aphasia Examination (BDAE), Boston Naming Test (BNT), Curtiss–Yamada Comprehensive Language Evaluation–Receptive Subtests (CYCLE-R), the Pyramid and Palm Trees Test (PPT), the Motor Speech Evaluation (MSE), and portions of the Psycholinguistic Assessments of Language Processing in Aphasia (PALPA)	Patients were injected with 8 to 10 mCi of FDG, and 30 min of emission data were collected at t 30 to 60 min after tracer injection	Study is constrained by the relatively small number of patients within each PPA subgroup, which limits our precision in estimating the prevalence of Aβ amyloidosis in each PPA variant	Medium
Tetzloff et al. 2018 [21]	Eleven subjects fulfilling diagnostic criteria for agPPA and 20 subjects fulfilling diagnostic criteria for PPAOS	When directly comparing agPPA and PPAOS, the agPPA group exhibited greater rates of change in specific brain regions, including left Broca’s area, middle frontal gyrus, superior frontal gyrus, superior medial frontal gyrus, orbitofrontal cortex, anterior and middle cingulate, insula, supplementary motor area, and lateral parietal lobe compared to PPAOS. Additionally, increased rates of change were observed in various areas of the frontal and parietal lobes in agPPA compared to PPAOS in the voxel-level analysis, although these results did not reach statistical significance after correction for multiple comparisons and are presented at an uncorrected threshold of *P* < 0.001	Western Aphasia Battery (WAB), The Token Test, Boston Naming Test, The Northwestern Anagram Test (NAT), Montreal Cognitive Assessment Battery (MoCA), Frontal Assessment Battery (FAB),Clinical Dementia Rating Scale sum of boxes (CDR-SB), Neuropsychiatric Inventory (NPI-Q), Movement Disorders Society sponsored revision of the Unified Parkinson’s Disease Rating Scale (MDS-UPDRS)	459 MBq of FDG (range 367–576 MBq) and PET acquisition occurred after a 30-min uptake period	A limitation was the small number of agPPA patients	High
Matias-Guiu et al. 2021	118 participants (31 patients with nfvPPA, 11 with svPPA, 45 with lvPPA, and 31 healthy controls)	Discrimination between patients with PPA and controls was 91.67%, with 77.78% discrimination between PPA variants	Comprehensive neuropsychological assessment, Addenbrooke's Cognitive Examination III, Corsi block-tapping test, Trail Making Test, Symbol Digit Modalities Test, Stroop Color-Word Interference Test, Rey–Osterrieth Complex Figure (copy and recall), Visual Object and Space Perception Battery, and Tower of London. Language was assessed using a battery of tasks, including picture naming, action naming, word-picture matching, action-verb matching, synonym judgment, semantic association, initial phoneme deletion	PET images were acquired following the European guidelines for brain FDG-PET imaging [2]	The number of svPPA patients was smaller than the groups for the other PPA variants	Medium
Madhavan et al. 2013 [23]	27	Atrophy and hypometabolism was observed in lateral temporoparietal and medial parietal lobes, left greater than right, and left frontal lobe in the logopenic group. The logopenic group showed greater left inferior, middle and superior lateral temporal atrophy (inferior *p* = 0.02; middle *p* = 0.007, superior *p* = 0.002) and hypometabolism (inferior *p* = 0.006, middle *p* = 0.002, superior *p* = 0.001), and less right medial temporal atrophy (*p* = 0.02) and hypometabolism (*p* < 0.001), and right posterior cingulate hypometabolism (*p* < 0.001) than dementia of the Alzheimer’s type	​​Mini-Mental State Examination, Boston Naming Test and Auditory Verbal Learning Test	FDG (average = 540 MBq; range = 366–399 MBq, uptake period = 30 min)	There was some age difference across groups	Medium
Matias-Guiu et al. 2014	35 patients total - 12 were classified as agrammatic variant, 4 were classified as semantic variant, 17 were classified as logopenic variant and 2 patients were unclassifiable	The clinical progression of PPA varies depending on the specific variant. In the agrammatic variant, reduced metabolism in the left anterior temporal region is associated with motor neuron disease, while hypometabolism in the medial frontal area is linked to atypical parkinsonism. Furthermore, the risk of progression in PPA may be influenced by factors such as the PPA subtype, the hemisphere predominantly affected, and the level of education	Evaluation of cranial nerves, motor and sensory systems, coordination, gait, and primitive reflexes, Harris’ test, Interview for Deterioration in Daily Living Activities in Dementia (IDDD) and Functional Activities Questionnaire (FAQ), Mini-Mental State Examination and Addenbrooke’s Cognitive Examination, Boston Naming Test, “Cookie Theft” picture from Boston Diagnostic Aphasia Examination, and language subtests of the Barcelona Test, The Progressive Aphasia Severity Score (PASS)	FDG (5 mCi) was administered intravenously 30 min before acquisition of images	There was a small sample size and the duration from the initial clinical presentation of PPA to the emergence of a second symptom was approximated based on medical assessments	Low
Matias-Guiu et al. 2015	33 PPA patients and 11 controls	Inter-rater agreement was found to be moderate for visual analysis (with a Fleiss' kappa value of 0.568) and substantial for statistical analysis (with kappa values ranging from 0.756 to 0.881). Agreement among raters was generally good for all three variants of PPA, except in the case of the nonfluent/agrammatic variant when using visual analysis When it came to diagnosing PPA, each rater demonstrated high sensitivity and specificity, with an average of 87.8% sensitivity and 89.9% specificity for visual analysis and 96.9% sensitivity and 90.9% specificity for statistical analysis employing global mean normalization. In the case of cerebellar normalization, sensitivity remained high at 88.9%, and specificity was 100%	The analysis of statistical images was conducted through two different approaches. In the first group, raters visually examined standard FDG PET images (referred to as visual analysis). Meanwhile, the second group of raters performed their analysis on images that had undergone statistical processing using the voxel-based brain mapping analysis technique, utilizing the Statistical Parametric Mapping software (SPM8) developed by The Wellcome Trust Centre for Neuroimaging at the Institute of Neurology in London, UK (referred to as the analysis of SPM maps)	FDG (185 MBq) was administered intravenously 30 min before images were taken	There were fewer cases with PPA-S patients	High

*PPA* primary progressive Aphasia, *FDG* 2-deoxy-2-[18F]fluoro-D-glucose, *PET* positron emission tomography, *AOS* apraxia of speech, *PFA* progressive fluent aphasia, *CASP* critical appraisal skills programme, *agPPA* agrammatic primary progressive aphasia, *PPAOS* primary progressive apraxia of speech, *nfvPPA* nonfluent variant primary progressive aphasia, *svPPA* semantic variant primary progressive aphasia, *PPA-S* primary progressive aphasia semantic subtype

## Results

After the initial search through databases, 98 articles were gathered to review. Of those papers, 67 were excluded based on the abstracts and titles. With the remaining 31 papers, seven articles met the PICO criteria and were included in this review. From the 98 articles, 44 papers were removed because of their focus on CSF biomarkers and amyloid PET, 29 papers were removed because of their focus on only AD, five papers were removed because of their focus on progranulin mutations rather than the diagnostic ability of FDG-PET, two papers focused on amyotrophic lateral sclerosis, seven papers used tau PET, three focused on corticobasal syndrome, and one paper was removed because of its focus on Huntington’s Disease.

**Fig. 3:**
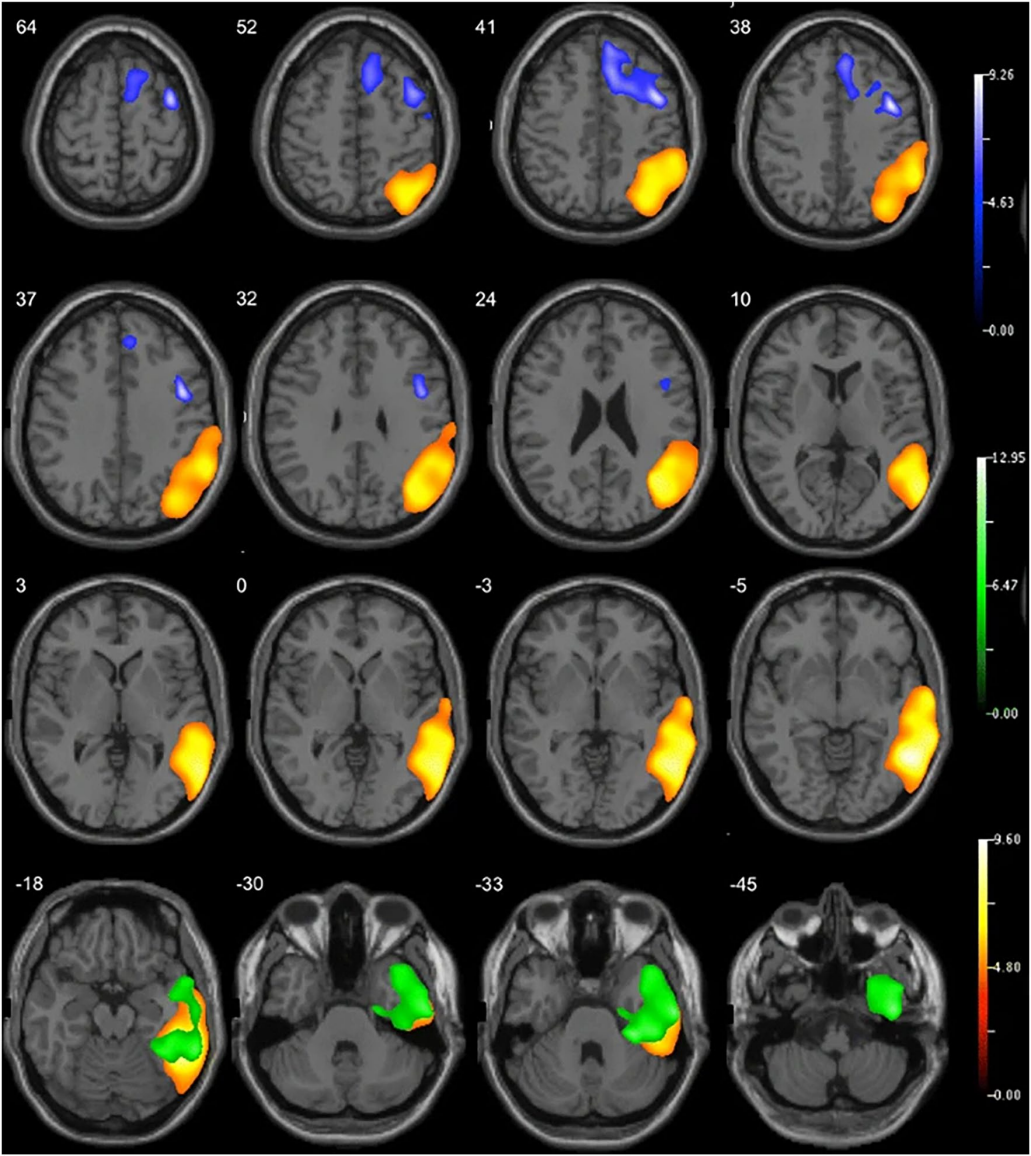
Delineation of hypometabolic regions at [^18^F] fluorodeoxyglucose positron emission tomography (FDG-PET) which can aid in differentiation subtypes of primary progressive aphasia. The blue areas indicate the nonfluent/agrammatic subtype, green indicates the semantic subtype, and yellow represents the logopenic subtype. Reproduced with permission from Matías-Guiu et al. [19]

Most FDG-PET studies showed alterations in regional metabolism among PPA patients, with distinctive imaging patterns among the three PPA subtypes. A visual example can be seen in Fig. 3. The reviewed studies collectively highlight the utility of FDG-PET in identifying distinct metabolic patterns associated with different PPA subtypes, though they also reveal some discrepancies. Josephs et al. (2010) emphasized early brain changes detectable by FDG-PET, focusing on patterns of hypometabolism without using control subjects, which limited their comparative insights. Rabinovici et al. (2008) provided a more detailed account, showing distinct hypometabolism patterns specific to PPA subtypes, such as reduced metabolism in the left temporoparietal region for LPA, left frontal area for PNFA, and left anterior temporal region for SD, with significant left-sided hypometabolism (*p* < 0.005). Tetzloff et al. (2018) noted greater rates of metabolic change in agPPA compared to PPAOS, particularly in frontal and parietal lobes, although these results lacked statistical significance after correction for multiple comparisons. Matias-Guiu et al. (2021) demonstrated high discrimination rates between PPA patients and controls (91.67%) and among PPA variants (77.78%), yet faced challenges due to smaller svPPA patient numbers. Madhavan et al. (2013) observed specific patterns of atrophy and hypometabolism in the logopenic group, emphasizing more pronounced effects in the left hemisphere compared to Alzheimer's type dementia. Matias-Guiu et al. (2014, 2015) further corroborated these findings, noting significant inter-rater agreement and high diagnostic sensitivity and specificity, although fewer cases with PPA-S patients were a limitation.

Overall, the SD subtype exhibited hypometabolism in the left thalamus, left inferior temporal gyrus, and the fusiform gyrus. PNFA demonstrated bilateral hypometabolism in the caudate nuclei, left hemisphere, thalamus, middle and superior temporal gyri, insula/inferior frontal gyrus, pars opercularis, lateral orbital gyrus, and middle frontal gyrus. Although the LPA variant had some similar regions of hypometabolism with AD, it has also showcased hypometabolism in the lateral temporoparietal and medial parietal lobes and left frontal lobe, differing from classical AD due to unique atrophy patterns. Concurrent observations of severe hypometabolism in the left temporal areas, with a more prominent decrease in left parietal activity compared to the left temporal lobe, were evident across all PPA variants. Patients showing glucose hypometabolism in the left angular, supramarginal, and posterosuperior temporal gyri areas underscores these regions' critical role in the development of aphasia. These studies consistently support the capability of FDG-PET to differentiate PPA subtypes, though variations in sample size, patient subgroup representation, and analytical methods underscore the need for more standardized approaches and larger cohorts to refine diagnostic precision.

## Discussion

### Differential diagnosis with PPA subtypes

Currently, the diagnosis of PPA is based on consensus guidelines such as the Gorno-Tempini criteria. However, the diverse presentations of PPA necessitate the inclusion of biomarkers for more accurate diagnosis [15]. FDG-PET shows distinct spatial metabolic patterns which can aid in distinguishing between various forms of dementia. A 2021 study conducted by Minoshima et al. demonstrated the ability to quantify disease progression in PPA by evaluating the atrophy rate using FDG-PET over time. This study demonstrates the ability of FDG-PET to highlight the specific locations affected by each subtype while clarifying or excluding the diagnosis of PPA in cases with clinically unclear presentations [16]. FDG-PET has also shown efficacy in detecting neuronal activity changes [17]. By analyzing various FDG-PET uptake patterns and parameters, PET offers an objective, non-invasive method for diagnosing neurodegenerative diseases [17].

This paper examined prior studies that explore the utilization of FDG-PET in differentiating PPA types. In this analysis, the effectiveness of FDG-PET in visualizing metabolic dysfunction has been mixed. One study, which involved 24 patients exhibiting a mix of either fluent and non-fluent aphasias demonstrated FDG-PET's utility in detecting brain hypometabolism changes by observing how FDG-PET hypometabolism patterns align with clinical classifications related to fluency [18]. After running the study, raters were assigned to label patients with PNFA, SD, or LPA, where they agreed upon approximately 70% of the labels [18]. Classifications into subtypes by raters were based on qualitative and quantitative data such as cross-modality deficits, word-finding difficulties, agrammatism, semantic and/or phonological errors, the presence of at least two of the disorder's features, as well as the operational definitions, defined by researchers of each respective study, for each subtype [18]. As part of the visual assessment, z score averages were taken for specific areas of interest such as the right and left lateral frontal, medial frontal, temporal, lateral parietal, medial parietal, and occipital cortices with a z score greater than 2 were recognized as significant. The main dispute revolves around the need to assess the frequency of short speech productions between complex sentences as a crucial factor for distinguishing between fluent and non-fluent aphasia. Regarding imaging findings, apraxia of speech was closely linked to hypometabolism in the superior frontal and supplementary motor cortex, while non-fluent aphasia was associated with the posterior inferior frontal lobe or Broca's area. Although this study demonstrated the presence of notable distinctions between the patterns of hypometabolism for fluent and nonfluent aphasias, the results suggest that there is still a need for uniformity about guidelines for all of the variants. Matias-Guiu et al. (2015) reported visual analysis agreement for the non-fluent subtype as most challenging between the raters, albeit with high agreement among other subtypes [18, 19]. In a similar study by Matias-Guiu et al. (2015), there was high diagnostic sensitivity and specificity of FDG-PET in PPA among raters, those in charge of diagnosing PPA using FDG-PET, with sensitivity averaging at 96.9% and specificity at 90.9% for statistical analysis. Visual analysis was demonstrated to be weaker with an average sensitivity of 87.8% and average specificity of 89.9%. Comparing the different methods of visual and statistical analysis, statistical analysis methods with FDG-PET show greater utility with diagnosing PPA variants because of the high inter-rater agreement and diagnostic accuracy [19].

Given the ambiguity in distinguishing features among different PPA subclasses, one approach taken by researchers is to center studies on a single PPA type, comparing it with other PPA types and control groups. For instance, a study by Tetzloff et al. focused on the agrammatic variant (agPPA) [21]. As agPPA progresses, neurodegeneration extends beyond the language networks, elucidating motor control weakening. Tetzloff et al. deduced a correlation between the disease’s progression and FDG-PET markers such as decreased metabolism, atrophy in gray matter within the middle and superior frontal gyri, premotor and motor cortices, medial temporal lobe, insula, basal ganglia, and brainstem. There was a particularly notable correlation with the increased atrophy in the left frontal lobe. Compared to control groups, the agPPA group displayed a distinct FDG-PET metabolism decrease, primarily in frontal areas [21].Comparing agPPA and primary progressive apraxia of speech (PPAOS) to controls also reveals that the two syndromes' patterns of advancement differ significantly, with PPAOS exhibiting more focused patterns of progression and agPPA displaying more diffuse progression throughout the language network. However, there was also overlap between the two syndromes. Due to the overlap of hypometabolism patterns and advancement within the same brain areas, the differential diagnosis continues to be difficult to parse with the PPA variants.

### Right vs left hemispheric involvement

An ongoing discussion in the field revolves around the 'right-sided' variant of SD and FTD, as described by Kumfor et al. [26]. Since the left and right hemispheres are responsible for different functions, understanding the relationship between neurodegeneration and hemispheric involvement has significant implications for clinical diagnosis and patient management. Left hemispheric involvement is seen in most PPA subtypes, such as SD, LPA, and PNFA. This region is associated with language processing, leading to symptoms such as impaired word meaning, object recognition, and speech production. On the other hand, the right-sided variant of SD involves the right temporal lobe and is associated with non-verbal deficits, such as impaired facial recognition, emotional processing, and social cognition [26]. The various hypometabolism patterns can be crucial in differential diagnosis, especially because misdiagnosis is likely with the atypical presentation of right-sided neurodegeneration. Targeted therapeutic plans can be used such as social cognition and emotional processing interventions for those with right-sided SD and language and speech therapy for those with left-sided involvement.

### Distinguishing between PPA and Alzheimer’s pathology

**Fig. 4:**
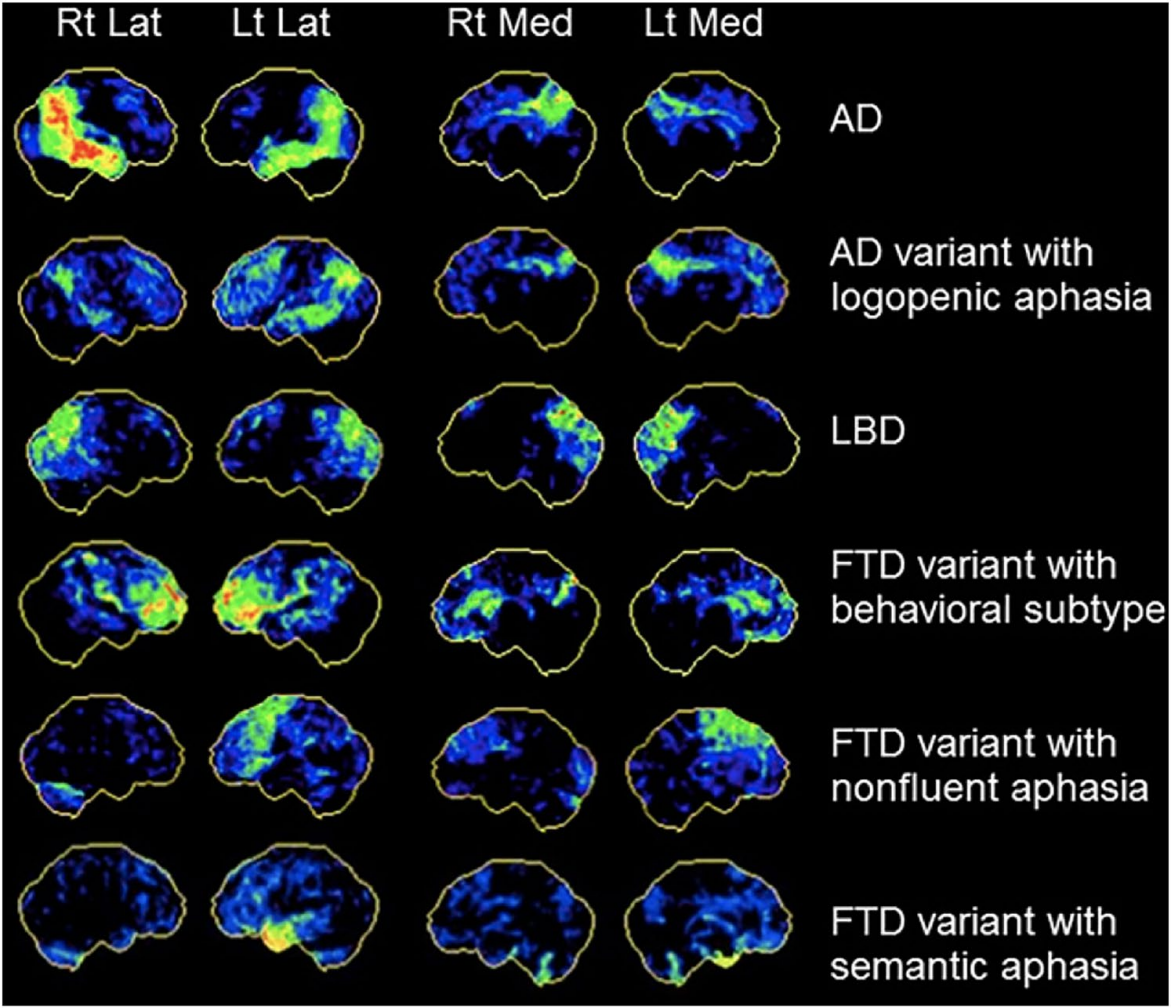
18F-FDG PET scan patterns for focal-onset dementias using Neurostat 3D-SSP where *Lat* lateral, *LBD* Lewy body dementia, *Lt* left, *Med* medial, *Rt* right. Reproduced with permission from Taswell et al. [20]

Another important challenge in diagnosis lies in differentiating the PPA subtypes from AD. PPA is often an early indicator of potential dementia development, with SD correlating to FTD and LPA considered an atypical AD presentation [18]. Autopsy findings revealed that around one-third of PPA patients, specifically those with LPA, demonstrated Alzheimer’s pathology [22]. The overlap of hypometabolism was evident in the medial parietal area, a hallmark of AD [18]. An example of the FDG metabolism can be seen in Fig. 4. However, in a study by Rabinovici et al., PPA patients showed greater asymmetric FDG uptake in language areas compared to AD patients [22]. In another study, the lateral temporoparietal and medial parietal lobes, and the left frontal lobe in PPA patients exhibited more pronounced atrophy and hypometabolism compared to patients with AD [22]. The similar hypometabolism patterns between LPA and AD underscores the importance of distinguishing between LPA and other PPA subtype [22, 24].

### Limitations

Non-English studies were excluded from the comprehensive literature review, which may introduce language bias and potentially limit the inclusiveness and representativeness of the analysis regarding FDG-PET's value in diagnosing PPA subtypes.

The principal limitation of these studies is the small PPA patient sample size. Furthermore, another major current challenge lies in identifying PPA patients without overlapping AD or Pick’s Disease pathologies. Additionally, with the current literature, there is no gold standard to accurately compare studies. Due to the lack of specific criteria or a reference, there are limited ways to quantify the effectiveness of FDG-PET. This served as a limitation for this review and also the studies included. Most studies focused on simple objective metrics, not looking at qualitative aspects or more complex quantitative measures.

Another limitation of this review is that most studies are done in conjunction with other clinical exams and imaging methods, such as MRI and amyloid PET. It is difficult to isolate the efficacy of FDG-PET individually when most clinics use multiple diagnostic techniques in conjunction.

Early PPA diagnosis can significantly aid in symptom management and treatment exploration. However, despite FDG-PET's utility, analyzing the differential diagnosis remains challenging due to overlapping impaired regions and the lack of uniform hypometabolism patterns among PPA variants, leading to common misdiagnosis or delayed accurate diagnoses. Awareness and knowledge of the various uptake patterns will help image interpreters and clinicians offer a more accurate diagnosis. Recognizing the nuances between different PPA subtypes, such as the left-sided hypometabolism versus the right-sided hypometabolism in certain SD cases can help improve determining the prognosis. Clinicians can use FDG-PET scans to tailor treatment strategies that target the specific cognitive deficits associated with each PPA subtype. Additionally, early identification of PPA subtypes can lead to enrollment in appropriate clinical studies and trials, possibly leading to better elaboration of pathways and treatment evaluation.

While FDG-PET has been proven useful in the diagnosis of PPA, multimodal imaging plays a crucial role as FDG-PET is often used alongside other imaging techniques. Combining different modalities, can enhance diagnostic accuracy by providing complementary information about brain structure, function, and metabolism. By integrating data from multiple imaging modalities, clinicians gain a comprehensive understanding of the underlying neurodegenerative processes.

## Conclusion

Neuroimaging studies of PPA patients underscore FDG-PET scan's promising role as a diagnostic modality, especially in distinguishing PPA subtypes. The significance of FDG-PET in PPA diagnosis is highlighted further given the challenge of accurate diagnosis and predicting the future onset of FTD and AD. Physicians should exercise diligence when reporting FDG-PET results in patients with language issues. Future FDG-PET studies with larger patient populations and well-designed cohorts of PPA patients can enhance our understanding of the disease and its related subclasses. Moreover, this knowledge could potentially unveil opportunities for therapeutic developments and evaluations for PPA.
